# The Health of Towns as Influenced by Defective Cleansing and Drainage; and on the Application of the Refuse of Towns to Agricultural Purposes; Being a Lecture Delivered at the Russell Institution, May 5, 1846

**Published:** 1847-01

**Authors:** 


Art. IV.—*The Health of Towns as influenced by defective Cleansing and Drainage; and on the application of the Refuse of Towns to Agricultural Purposes: being a Lecture delivered at the Russell Institution, May* 5, 1846. By William A. Guy, M.B. Cantab., &c. &c.—*London,* 1846. 8vo, pp. 48.

Dr. Guy, in this pamphlet, very lucidly sets forth the advantage and economy of a better system of civic drainage. The gross value of the manure annually wasted amounts, he thinks, to 10,000,000*l.*; the health-tax inflicted upon the population by sewerage defects he estimates, for the United Kingdom, at nearly 20,000,000*l.* If we halve and quarter these sums, after the approved method of estimating an heiress's fortune, there remains a very handsome sum; and we certainly think that several millions sterling are annually wasted in the way Dr. Guy points out, although we hardly think his numerical estimate will be received as being mathematically accurate. Much more investigation is required on the points Dr. Guy moots in this essay, before ACTION will arise. The public will thank him, we trust, for being willing to lead in the van in an attempt at great social improvement.

